# Topological and statistical analyses of gene regulatory networks reveal unifying yet quantitatively different emergent properties

**DOI:** 10.1371/journal.pcbi.1006098

**Published:** 2018-04-30

**Authors:** Wilberforce Zachary Ouma, Katja Pogacar, Erich Grotewold

**Affiliations:** 1 Molecular and Cellular Imaging Center (MCIC), Ohio Agricultural and Research Development Center (OARDC), Ohio State University, Wooster, OH, United States of America; 2 Department of Biochemistry and Molecular Biology, Michigan State University, East Lansing, MI, United States of America; Ottawa University, CANADA

## Abstract

Understanding complexity in physical, biological, social and information systems is predicated on describing interactions amongst different components. Advances in genomics are facilitating the high-throughput identification of molecular interactions, and graphs are emerging as indispensable tools in explaining how the connections in the network drive organismal phenotypic plasticity. Here, we describe the architectural organization and associated emergent topological properties of gene regulatory networks (GRNs) that describe protein-DNA interactions (PDIs) in several model eukaryotes. By analyzing GRN connectivity, our results show that the anticipated scale-free network architectures are characterized by organism-specific power law scaling exponents. These exponents are independent of the fraction of the GRN experimentally sampled, enabling prediction of properties of the complete GRN for an organism. We further demonstrate that the exponents describe inequalities in transcription factor (TF)-target gene recognition across GRNs. These observations have the important biological implication that they predict the existence of an intrinsic organism-specific *trans* and/or *cis* regulatory landscape that constrains GRN topologies. Consequently, architectural GRN organization drives not only phenotypic plasticity within a species, but is also likely implicated in species-specific phenotype.

## Author summary

The translation of genotype to phenotype is a tightly regulated process that is mediated by specific interactions between a variety of cellular components. Central to this is the transcription of genes, a process regulated by proteins that bind DNA, including the transcription factors (TFs). Gene regulatory networks (GRNs) describe the web of protein-DNA interactions essential for the regulation of biological pathways and developmental processes. Here, we describe fundamental properties of the architectural organization of eukaryotic GRNs. Using protein-DNA interaction data derived from the budding yeast, the fruit fly, *Caenorhabditis elegans*, and *Arabidopsis*, we determine that GRNs are scale-free, wherein a majority of TFs bind comparatively fewer target genes, while a small number of TFs bind a large number of genes. Further, we show that the scale-free connectivity power-law coefficient is organism-specific, suggesting differential wiring patterns across GRNs. We then capitalize on the organism-specific connectivity to develop a mathematical framework to predict the number of total interactions in the complete GRNs, important for understanding how the expression of all genes in an organism is regulated, and for experimental design purposes. Finally, we demonstrate numerically and by simulations that subnetworks sampled from scale-free GRNs are scale-free, as long as edges are sampled. This finding has important real-life implications to infer properties of a network with limited experimental data.

## Introduction

Complex systems are formed by large numbers of components organized into networks, and modelled by graphs in which nodes are connected by edges. Network architecture is established by topological and statistical analyses, ultimately leading to inference of functional roles played by the nodes in the network, and prescribed by the observed architecture. Efforts to infer information flow, which ultimately leads to functional outputs, have been applied to different types of networks, including social communication, electrical power [1], and biological [2–10]. A general characteristic of many real-world networks is their scale-free topologies, which exhibit a node degree distribution that can be described with a power law function:

$$P(k)=Ck^{-\alpha }$$ (1)

where *P(k)* is the probability of a randomly selected node having degree *k* (that is *k* connections), and *α* is the power law scaling exponent (hereafter referred to as the exponent). The constant *C* is a Riemann’s zeta function that normalizes the power law probability distribution, such that:

$$\sum_{k=1}^{\infty }P(k)=1$$ (2)

In scale-free networks, most nodes have comparatively few interactions manifested as a lower degree, while a small number of nodes, the ‘hubs’, have a higher degree [11, 12]. This scale-free connectivity distribution is observed at different levels of biological organization ranging from the cellular and molecular, to the ecological level. Gene regulatory networks (GRNs), characterized by the interaction of a specific type of proteins, the transcription factors (TFs) with the regulatory DNA regions in the genes that the TFs control, provide excellent examples of molecular-level scale-free networks [2, 6, 10, 13–15]. GRNs can be represented by directed graphs in which the edges have a polarity, because a TF can bind to the regulatory region of a gene (which may encode for another TF) and modulate its expression, but not vice versa. Thus, GRNs can be visualized from the perspective of incoming connectivity (i.e., how many TFs bind to a specific gene regulatory region), or from the outgoing connectivity perspective (i.e., how many regulatory regions does a TF recognize). The molecular tools available to identify incoming and outgoing connectivity are different. Incoming connectivity is usually mapped using gene-centered approaches such as yeast one-hybrid (Y1H) assays [16], and outgoing connectivity is evaluated by TF-centered approaches such as chromatin immunoprecipitation (ChIP)-based (e.g., ChIP-Seq and ChIP-chip) [17] or DNA affinity purification sequencing (DAP-Seq) methods [18]. While the integration of results derived from gene- and TF-centered procedures should ultimately converge into the same GRN, much of the unbiased data available today derives from TF-centered approaches, providing a much clearer perspective of outgoing connectivity. We anticipate that the advent of new experimental approaches to map PDIs and place them in a biological context will permit to explore the convergence of incoming and outgoing connectivity in many organisms.

**Fig 1:**
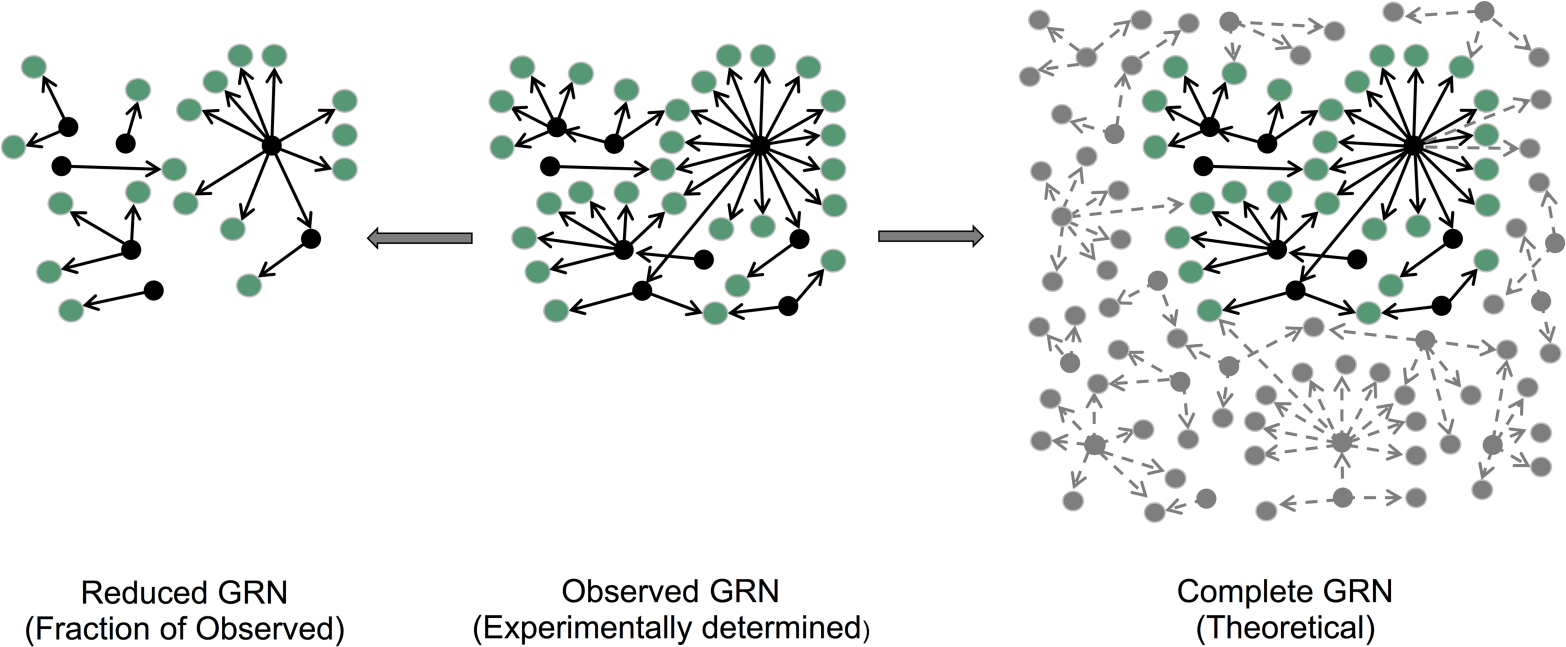
Framework for sampling and predicting properties of complete GRNs. The subnetwork on the left depicts a reduced GRN sampled from the observed GRN (center) whose properties can be used to infer the properties of complete GRN depicted on the right. Black and grey nodes denote TFs and non-TF-coding genes, respectively. Dashed lines represent possible PDIs in complete GRNs that are yet to be identified, but are predicted based on the scale-free property of observed GRNs.

Organismal phenotypic plasticity is driven in part by the underlying GRNs [19–21]. Therefore, the reconstruction and topological analysis of GRNs provides an excellent opportunity for elucidating molecular mechanisms that drive phenotypic plasticity. However, despite significant research in this area, little is known with regards to whether GRNs from different organisms have similar emerging properties that only depend on node number, or whether properties such as network connectivity, manifested for example in the exponent of the power law, are unique to each organism. Experimentally, most studies will be able to provide at best an observed network, which corresponds to a subset of the complete true network (Fig 1). It is unclear to what extent properties of the observed network can be used to infer properties of the complete network (Fig 1). Conversely, several studies have investigated the properties of subnetworks, starting from synthetic or natural networks. The conclusions derived from these studies depend on the sampling method used [22–24]. For example, it was argued that randomly selected subnets of scale-free networks are not scale-free themselves, and that therefore inferences about the complete network had to be treated with caution [25]. However, the node sampling methodology used in that study results in a loss of degrees because, by targeting nodes rather than edges, all the edges associated with a node are lost, resulting in the enhanced decrease in degrees. In addition, that study did not model the stochasticity inherent in the sampling process, thereby not capturing the possible range of degree exponents that a subnetwork can take. As described here, sampling edges while accounting for stochasticity gives a very different result.

In our study, we take four representative model organisms that represent major eukaryotic evolutionary groups (the yeast *Saccharomyces cerevisiae*, the worm *Caenorhabditis elegans*, the fruit fly *Drosophila melanogaster* and the flowering plant *Arabidopsis thaliana*) and for which a wealth of PDI data is publicly available, to reconstruct GRNs followed by network connectivity analysis. Following simulations and rigorous statistical analyses, we demonstrate that GRNs exhibit organism-specific scale-free connectivity, revealed by distinct exponents of the out-degree. Further, we show that the observed coefficients are unbiased estimates of exponents derived from the degree distribution of the inferred complete GRNs. As a result, we apply a Monte-Carlo simulation approach for the estimation of the number of PDIs in complete GRNs. To provide an interpretation of the out-degree exponent, we employ ‘inequality’ analyses using Lorenz curves. We show that the exponents describe the relationship between the proportions of TFs binding to the corresponding fraction of the target genes. The resulting GRN topologies can therefore be classified as either ‘capitalistic’, exemplified by the presence of a handful of hub TFs that bind a significant and disproportionate number of target genes, or ‘socialistic’, in which TFs bind a near corresponding proportion of targets. Collectively, these observations demonstrate the utility of the observed GRNs in predicting properties of complete GRNs, with important implications for understanding the complex regulatory repertoire of eukaryotic organisms.

## Results

### GRNs exhibit scale-free, organism-specific connectivity

**Fig 2:**
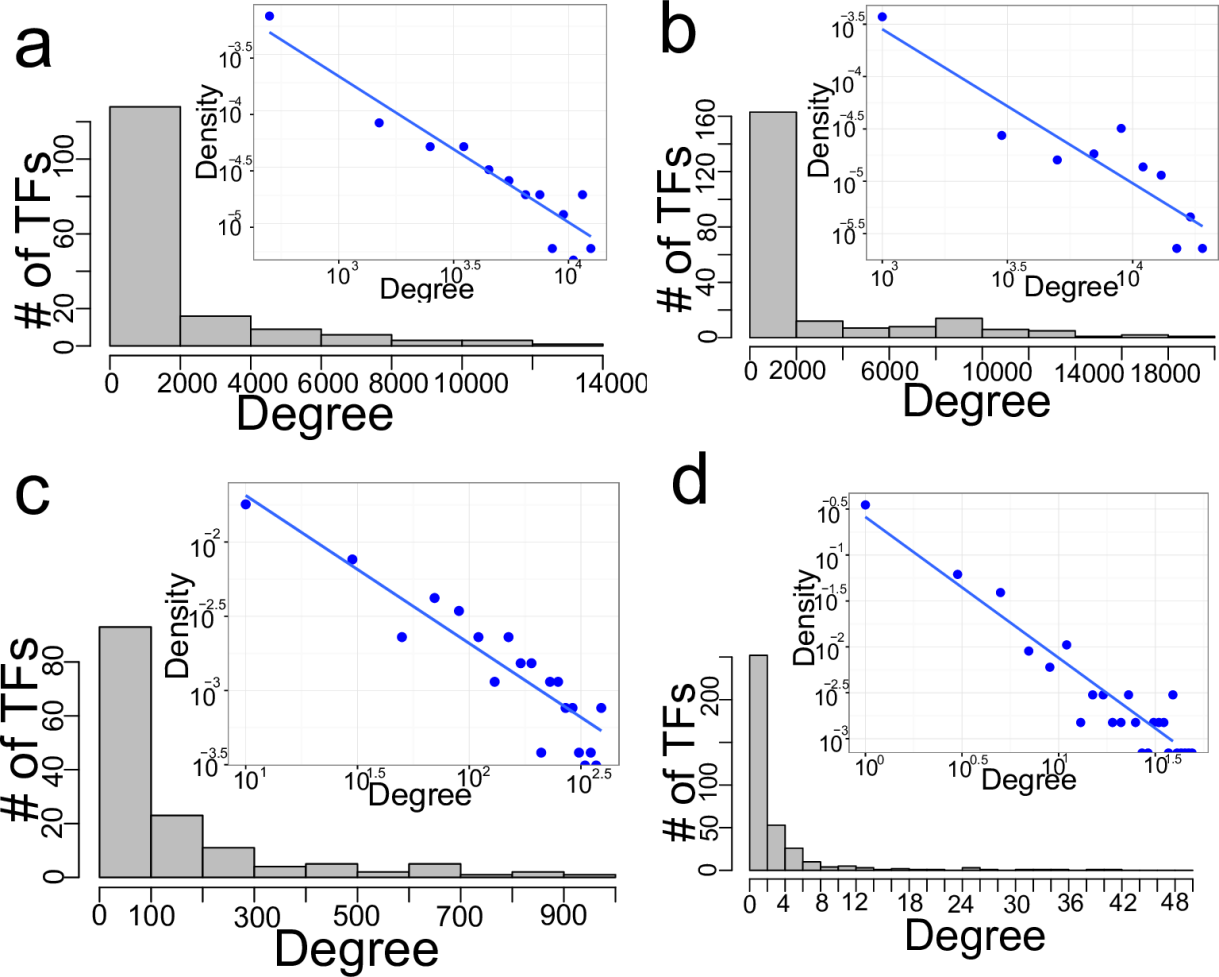
Out-degree connectivity of GRNs. Histograms depicting out-degree distribution corresponding to GRGs of *D*. *melanogaster* (a), *C*. *elegans* (b), *S*. *cerevisiae* (c), and *A*. *thaliana* (d). Inset: binned density plots of the respective degree distributions on log-log scale.

We constructed GRNs using all available experimentally determined PDIs derived from ChIP-Seq, ChIP-chip, and yeast one-hybrid assays (Table 1). To determine the connectivity of these observed GRN, we enumerated the target genes bound by each TF (out-degree, Fig 2), and the number of TFs binding each target gene (in-degree, S1 Fig). We observed that, in all four organisms investigated, a majority of TFs bind comparatively to few target genes (low degree TFs), while a small number of TFs bind to a large proportion of target genes (high degree TFs). A linear relationship of the probability density function on a log-log scale was observed (Fig 2, inset), indicative of the scale-free property of the interaction distribution. To unequivocally confirm the scale-free properties of the resulting observed GRNs, we implemented a formal statistical analysis framework consisting of the following steps: (i) Fitting node-degree distribution to a power law function and estimating the power law function exponent parameter (*α*) using the maximum likelihood approach; (ii) testing goodness-of-fit by comparing the fitted power law distribution and the empirical node degree distribution using the Kolmogorov-Smirnov (KS) *D* statistic; and (iii) performing pairwise model selection by comparing the fitted power law distributions to Poisson and exponential functions. A non-nested model selection approach that uses the Kullback-Leibler information criterion (Vuong’s closeness test) was employed for the pairwise model comparisons (see Methods for details). We observed a significant fit of power law functions on the out-degree distribution (Table 2), thereby confirming the scale-free nature of the observed GRNs. Further, model selection likelihood ratio tests comparing fitted power law and Poisson distribution functions (a descriptor of non-scale-free random networks) revealed that fitted power law distributions are significantly favored (Table 2). As anticipated, given the biased nature and insufficient sampling of most gene-centered PDI determination studies, in-degree distribution of the available experimental data could not be described by a power law function. Unexpectedly, the out-degree power law exponents were different for the observed GRNs obtained from the four organisms, with values of 4.12 for *C*. *elegans*, 3.04 for the fruitfly, 2.0 for yeast and 1.73 for *Arabidopsis* (Table 2, first row).

**Table 1:** Metrics from the observed GRNs.

Metric/ No. of	*C*. *elegans*	*D*. *melanogaster*	*S*. *cerevisiae*	*A*. *thaliana*
TFs in GRN (% TFs in the genome)	219 (23)	166 (16)	138 (46)	357 (15)
TFs in genome^a^	~ 934	~ 1052	~ 301	~ 2451
Target genes in GRN (% coding genes in the genome)	20,220 (99)	15,293 (90)	4,263 (71)	10,304 (37)
Genes in the genome	~ 20,470	~ 17,000	~ 5,000	~ 27,655
Nodes (TFs & target genes) in GRN	20,220	15,302	4,271	10,383
Interactions (PDIs) in GRN	464,258	229,615	26,091	17,038

^a^The total number of TFs in the genome was obtained from Reece-Hoyes, 2005 for *C*. *elegans*; FlyTF.org for *D*. *melanogaster*; de Boer & Hughes, 2011 for *S*. *cerevisiae*; and PlnTFDB for *A*. *thaliana*.

**Table 2:** GRNs are scale-free.

Parameter	*C*. *elegans*	*D*. *melanogaster*	*S*. *cerevisiae*	*A*. *thaliana*
Out-degree power law exponent (KS *p-*value)	*4*.*12 (0*.*700)*	*3*.*04 (0*.*820)*	*2*.*0 (0*.*500)*	*1*.*73 (0*.*738)*
Vuong’s statistic with Poisson model (*p-*value)	*3*.*30 (0*.*001)*	*5*.*00 (0*.*000)*	*3*.*50 (0*.*000)*	2.68 (0.08)
Vuong’s statistic with exponential model (*p-*value)	*4*.*00 (0*.*000)*	0.93 (0.350)	-0.73 (0.465)	*2*.*47(0*.*014)*

Power law fit, row 2: Power law function parameter values and Kolmogorov-Smirnov (KS) *D* statistic *P*-values (in parenthesis) obtained from fitting out-degrees on Power law function. Italicized values denote significant fit of out-degree at alpha greater than 0.1. Model selection, rows three and four: A pair-wise comparison between fitted out-degree power law and Poisson distribution (third row) and exponential distribution (fourth row). Pair-wise comparisons were performed using a non-nested likelihood ration test, the Vuong’s test. A positive Vuong’s statistic and *P-*value less than 0.05 indicate power law is favored over a competing model; negative statistic with a non-significant *P*-value (at alpha 0.05) denote insufficient evidence in favor of one models over the other.

To determine the difference between the empirical distributions of out-degrees for pairs of observed GRNs, the two-sample Kolmogorov-Smirnov (KS) test was employed, with the null hypothesis testing whether two samples have been drawn from the same distribution. We observed that pairs of out-degrees between organisms have distinct distributions, with the exception of *A*. *thaliana—S*. *cerevisiae* and *D*. *melanogaster—S*. *cerevisiae* comparisons (Table D in S1 Information).

**Fig 3:**
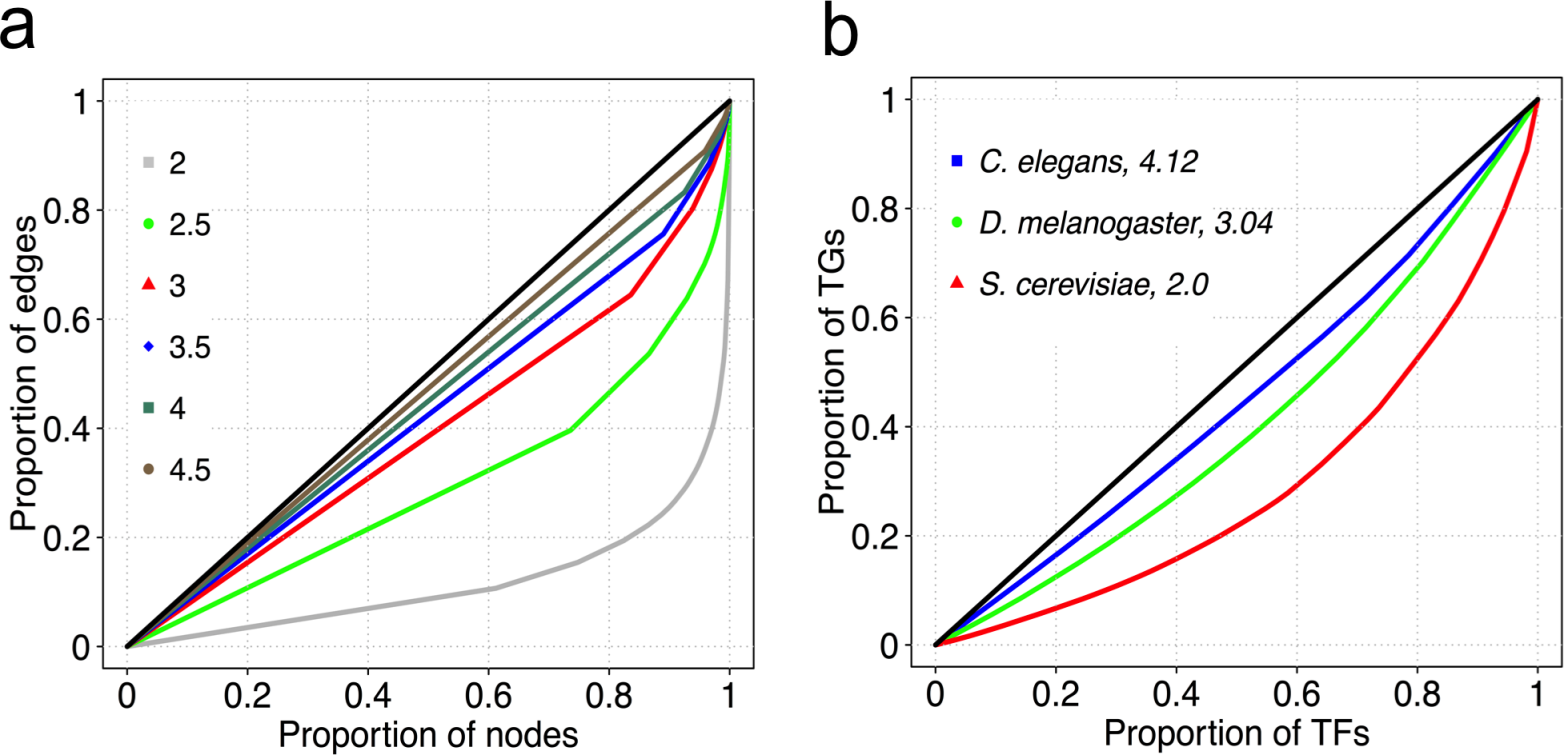
Lorenz curves. Simulated Lorenz curves for exponents ranging from 2 to 4.5 (a). Lorenz curves for out-degrees of observed GRNs (b). TGs: target genes.

To investigate the possible biological consequence of the different out-degrees in the scale-free topology of GRNs for the four organisms, we investigated how ‘inequality’ in TF-target gene binding distributions is affected by the power law degree exponent in the different GRNs, using Lorenz curves [26]. We ranked TFs based on increasing number of target genes and plotted the cumulative proportion of target genes as a function of the corresponding cumulative proportion of TFs. Interestingly, we observed an increase in degree ‘equality’ for each increase in the value of the exponent (Fig 3). This contrasts with a perfectly egalitarian distribution of degrees where all TFs have approximately the same degree, and for which the associated Lorenz curve becomes the diagonal of the plot, referred to as the line of equality. Thus, GRNs with smaller exponents, such as the *S*. *cerevisiae* GRN, have hub TFs that bind disproportionately more target genes, compared to the same number of hub TFs in GRNs with higher exponents (Fig 3). Indeed, we observed that the top 20% of TFs with the highest number of target genes in *S*. *cerevisiae* bind about 50% of the target genes. In *C*. *elegans*, a similar proportion (the top 20%) of TFs binds to 30% of target genes. In an egalitarian binding scenario, 20% of the top TFs would bind 20% of the target genes. Note that our analyses here and henceforth did not include *Arabidopsis* out-degrees due to the low number of target genes (37% of all coding genes) represented in the observed GRN (Table 1), corresponding to insufficient sampling of out-degrees.

Next, we investigated potential biases in the estimation of the exponents that might be driven by data sources. In this regard, we estimated exponents from subnetworks derived from specific experimental data source (ChIP-Seq, ChIP-chip or Y1H) and tissues type, where data is available. Investigating the influence of experimental data source in *D*. *melanogaster* GRN revealed similar exponents for the data source-specific subnetworks (Table A in S1 Information). For instance, with the exception of a slight increment of 0.32 for the ChIP-Seq-derived subnetwork, the exponents of ChIP-derived subnetworks are similar (rows 2, 3, and 4 of Table A). In the case of Y1H, it is evident that gene-centered approaches employed in building GRNs can inadvertently introduce bias due to insufficient sampling of out-degrees. Y1H contributed only 406 (~ 0.18% of total GRN) interactions to the *D*. *melanogaster* GRN and 136 TFs, indicating that on average one Y1H-derived TF binds 2 target genes (208/136)–an unlikely *in vivo* phenomenon. Indeed, fewer out degrees are sampled in Y1H because the technique depends on cloning promoters of target genes. Difficulties in cloning promoters, as well as the comparatively higher numbers of targets in a genome (unlike TFs), results in an underrepresentation of out degrees in Y1H-derived GRNs. TF-based techniques (ChIP-Seq and ChIP-chip) such are more attractive in construction of GRNs that capture the expected connectivity because of the near complete sampling of out degrees (targets). TF-centered approaches are however not immune from potential bias, primarily the inclusion of false-positives targets. The ChIP-Seq and ChIP-chip analysis pipelines attempt to account for the false positive rates by determining the false discovery rates (FDRs) in cases where biological replicates exist. It’s important to note that in our analysis, inclusion of the Y1H data did not result in a deviation of the power law exponent (compare rows 2 and 3 in Table A of S1 Information). Minimal deviations were also observed in the data-specific subnetworks of *C*. *elegans* (Table B) and *S*. *cerevisiae* (Table C).

Another potential bias in estimation of exponents is tissue- (or developmental) specific sampling of targets. To address this, we sampled a subnetwork from the *D*. *melanogaster* GRN PDIs derived from the embryo stage. The choice for *D*. *melanogaster* was largely due to availability of tissue-specific data. The analyses resulted in a subnetwork with 47 TFs, 178,224 PDIs, and a total of 15,016 nodes. Fitting a power law function on the ‘embryonic’ out-degrees resulted in an exponent of 3.10 and KS *P*-value of 0.86 (row 7 of Table A). It is worth noting that these deviations fall within the 95% prediction intervals of the expected range of exponents for complete GRNs (see next subsection on inference of properties of complete GRNs).

Taken together, these findings demonstrate that, while GRNs are characterized by the unifying scale-free network property as opposed to random degree distribution, the GRN connectivity is quantitatively organism-specific, suggesting intrinsic organismal properties that define TF binding landscapes.

### Inferring complete network scale-free properties from observed networks

The ‘observed’ GRNs described in the prior section correspond to a fraction of the ‘complete’ GRNs that remain to be experimentally determined. A fundamental question that this study intends to address is to what extent can the observed GRNs be used to infer properties of complete GRNs (Fig 1). The answer serves two main purposes: first, one goal of systems biology is to describe all system components and their associated interactions. Since current GRNs are incomplete, but are samples from complete yet unobserved GRNs, there is need to determine whether properties of current GRNs sufficiently describe properties of the intended complete GRNs. Second, decisions on whether additional experiments need to be performed will be based on the ability of the current GRNs in describing properties of complete GRNs, an important consideration in experimental design. Therefore, to determine whether complete GRNs are scale-free with organism-specific degree scaling exponents, we evaluated the distribution of degrees of nodes sampled from large populations of simulated node degrees whose power law exponents are known. We describe the approach below.

We implemented a Monte-Carlo (MC) simulation approach to generate large populations of simulated nodes, each population exhibiting a distinct and known power law exponent of the node degrees (see Note A in S1 Information for a detailed description on sampling). Briefly stated, we first generated three sets of a large number of simulated nodes (*n =* 10,000), each with population degree power law exponent, *α*_*pop*_, corresponding to the three observed exponents of 4.12, 3.04 and 2.00. In the MC simulation, the number of computationally-generated nodes significantly exceeded the number of TFs in any organism in order to model a theoretically large population of nodes, a condition required for the central limit theorem (CLT) to be applicable (see Note A in S1 Information). Next, we randomly drew nodes from each population (with replacement) to generate samples (*r =* 1,000) of different sizes, followed by estimation of the scaling exponent of each sample using the maximum likelihood method. The distribution of exponents for large sample sizes (*e*.*g*., *n* = 5,000) followed normality with their average corresponding to the population exponent (S2A Fig). As anticipated, we observed a marked deviation from normality coupled with increased variance whenever smaller samples (*n* < 30) were drawn (S2B Fig). To predict the range of exponents for the complete GRNs, we calculated prediction intervals (PIs) using standard deviation (SD) of their distribution derived from the MC sampling procedure. We specifically used the MC-derived SDs corresponding to the number of TFs in the genome to construct 95% PIs (Note A in S1 Information). We observed 95% PIs falling in the {3.51–4.73}, {2.76–3.32}, and {1.87–2.13} intervals for the starting degrees of 4.12, 3.04 and 2.00, respectively. Notably, the PIs for exponents of complete GRNs do not overlap, thereby underscoring the organism-specific nature of power law scaling exponents in GRNs of the organisms investigated here.

From this analysis, we conclude that, a scale-free observed GRN with exponent *α_obs_* is likely derived from a complete true GRN, which is also scale free with exponent *α_obs_* ± *c*, where *c* is the upper and lower bounds of the 95% PI. In the following section, we capitalize on the predicted exponents of complete GRNs to estimate the size of their corresponding complete GRNs.

### Estimating the number of PDIs in a complete GRN

There is a pressing need to infer properties of complete GRNs in order to capture the system-wide regulatory landscape of a particular organism. However, experimental limitations (such as challenges in generating TF-specific antibodies for ChIP, limitations in genome sequence and annotation) and lab-specific research questions have resulted in incomplete and often fragmented GRNs, whose properties may fail to adequately capture the intended entire regulatory repertoire. To address this challenge, we undertook a simulation approach to estimate the expected number of PDIs in the complete GRNs. Our method is predicated on the finding that the observed out-degree exponent of a GRN is an unbiased estimate of the respective complete GRN exponent. As a consequence, out-degrees of an observed GRN can be described as a random sample from a population of degrees corresponding to the number of TFs in the genome. In the observed GRNs, the number of interactions (*I_obs_*) is obtained by the summation of the out-degrees in the network, as follows:

$$I_{obs}=\sum_{i=1}^{n}k_{obs,i}$$ (3)

where *n* is the total number of TFs (number of out-degree nodes) in the observed GRN, and *k*_*obs*,*i*_ is the *i*^*th*^ observed out-degree value. We extend this framework to identify the number of interactions for a complete GRN (*I*_*comp*_), and posit that:

$$I_{comp}=\sum_{i=1}^{N}k_{comp,i}$$ (4)

where *N* is the total number of TFs in the genome and *k*_*comp*,*i*_ is the *i*^*th*^ out-degree value of the complete GRN (see Note B in S1 Information for a detailed description on derivation of simulated degrees). To test the feasibility and accuracy of the simulation approach, we estimated the actual number of interactions (*I*_*obs*_) for the observed GRNs. We determined that, on average, the number of PDIs estimated by the method was equal to the number of PDIs of the observed GRNs (Table 3, column 3; refer to Note B in S1 Information for description on hypothesis testing). We subsequently used this method to predict the number of interactions of the complete GRNs (*I*_*comp*_) based on the total number of TFs and genes that have been described in the organisms (Table 1). The maximum possible number of PDIs (upper bound) corresponds to the product of the total number of TFs and the total number of genes, as this would imply that every TF binds to every gene in the genome (Table 3, column 5). When we computed *I*_*comp*_ for the organisms we investigate here, we found that the budding yeast *S*. *cerevisiae* would have a total of ~60,000 PDIs, the fruitfly *Drosophila* has ~1.5M PDIs and the worm *C*. *elegans* has ~2M PDIs (Table 3, column 4). These estimates suggest that the number of observed PDIs represents ~45%, 14%, and 23% of the respective complete GRNs. When we compare the predicted PDI number of the complete GRNs with the maximum possible, we find that it is only 4% for yeast, 8% for *Drosophila* and 10% for *C*. *elegans* (Table 3, column 5), indicating that combined, TFs are sampling only a fraction of all the possible TF-target gene combinations. This observation contrasts the continuous network model that proposes *in vivo* binding of each TF to essentially all target genes in an organism.

**Table 3:** Estimation of number of interactions for complete GRNs.

Organism	Observed # of PDIs	Simulated observed # of PDIs ± SD (Z-test *P-*value)	Estimated # of PDIs for complete GRNs (Range of % of complete GRN observed thus far)	Maximum # of possible PDIs (Complete GRN as % of max. possible PDIs)
*D. melanogaster*	229,615	250,081.6 ± 15,638 (0.19)	1,585,528 ± 39,650 (14.1% - 14.9%)	17,884,000 (8.6% - 9.1%)
*C. elegans*	464,258	471,563.7 ± 14,376 (0.63)	2,010,004 ± 29,341 (22.8% - 23.4%)	19,118,980 (10.4% - 11.7%)
*S. cerevisiae*	26,091	30,070 ± 5,110 (0.44)	59,962 ± 7,131 (38.9% - 49.4%)	1,505,000 (3.5% - 4.5%)

The third column shows the accuracy of the simulation method in predicting the observed number of PDIs (hypothesis testing description in Note B in S1 Information); fourth column shows the estimated number of PDIs for theoretically complete GRNs; fifth column depicts maximum number of possible PDIs when all TFs in a genome bind all genes. SD: standard deviation.

### Subnetworks of scale-free networks are scale-free

Having demonstrated that complete GRNs are scale free, we set out to determine whether subnetworks of observed scale-free GRNs are equally scale-free. By sampling edges from observed GRNs, we mimic the experimental approach involved in constructing GRNs. Indeed, construction of GRNs largely involves identifying interactions (edges) between known cellular components (TFs and potential target genes). Below, we first show analytically followed by sampling, that subnetworks of scale-free GRNs are scale-free. When drawing edges from a GRN, the probability *Pr(i)* of a node *i* in the GRN becoming node i* in the subnetwork given that its edge has been randomly selected is dependent on node *i* degree, *k*_*i*_. This relationship is described by:

$$Pr(i^{*})=\frac{k_{i}}{k_{T}}$$ (5)

where *k*_*T*_ denotes the total number of out-degrees in a GRN. To sample subnetworks of different sizes, edges are sampled with probabilities *0<p<1*. Therefore, the probability of including node i* in the subnetwork when edges are sampled with a probability *p* is:

$$Pr(i_{p}^{*})=p\times \frac{k_{i}}{k_{T}}$$ (6)

When sampling subnetworks of specific sizes (*e*.*g*., half the observed GRN, where *p =* 0.5), *p* and *k*_*T*_ are constants in Eq 6, which can be rewritten as:

$$Pr(i_{p}^{*})=\frac{p}{k_{T}}k_{i}+\varepsilon$$ (7)

where *ε* is an error term accounting for the pseudorandom number generator (PRNG) since the PRNG algorithm is not strictly random but depends on an initial value, the seed. It is clear from Eq 7 that the probability of a node being included in the subnetwork, when its edges are sampled, is a linear function of the degree of the node being sampled. Thus, analytically, the degree distributions of a GRN and its associated subnetworks are similar.

**Fig 4:**
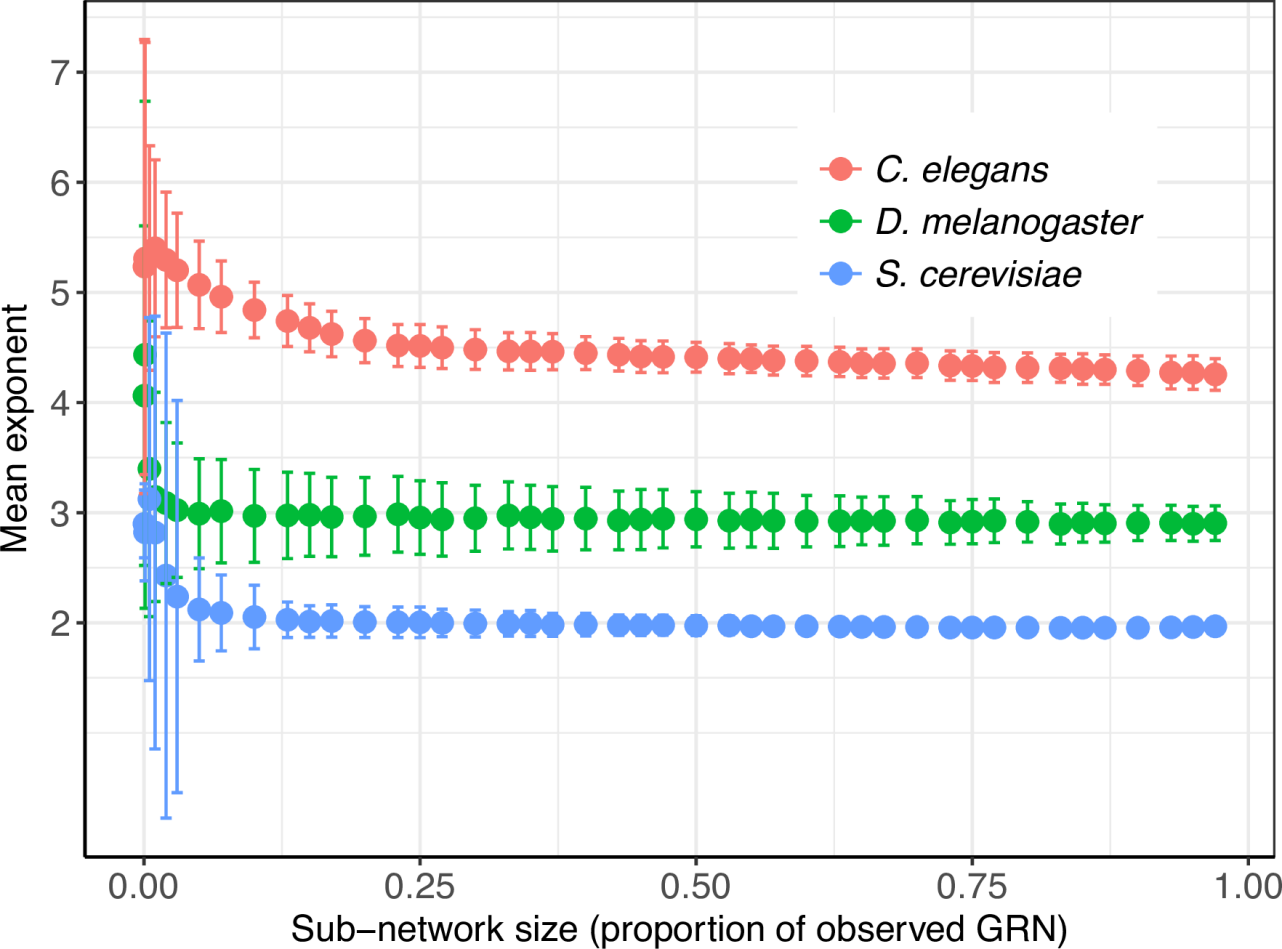
Sampling subnetworks. Distribution of exponents of subnetworks sampled from observed GRNs.

**Fig 5:**
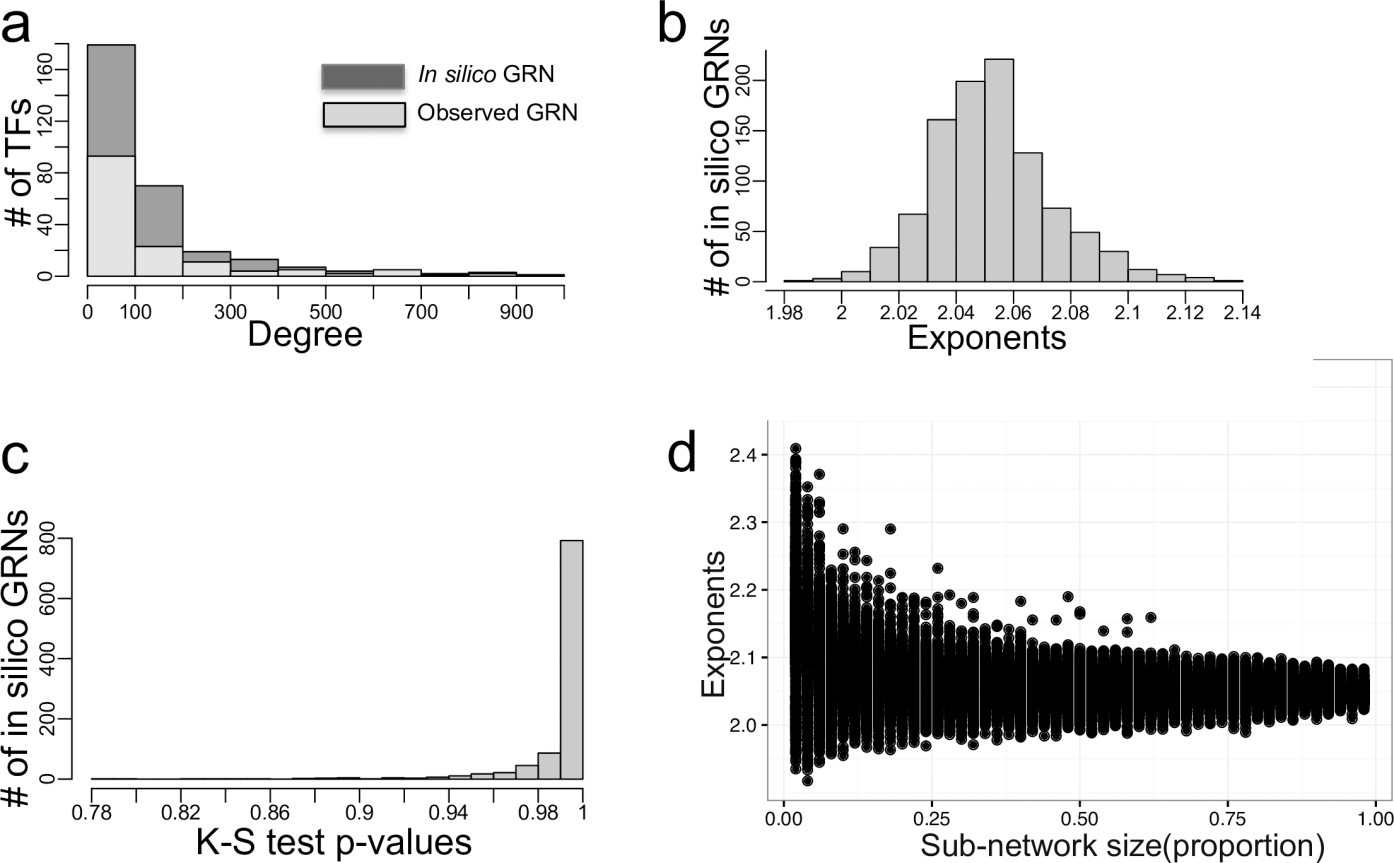
*In silico* GRNs. (a) Histogram of the out degree distribution of an *in silico* GRN and of an observed GRN corresponding to *S*. *cerevisiae*. (b) Distribution of the exponents for each of 1000 *in silico* GRNs. (c) Distributions of KS *P-*values of sub-networks sampled from an *in silico* GRN. (d) Distribution of exponents of sub-networks sampled from *in silico* GRN.

For computationally validating the aforementioned analytical procedure while accounting for stochasticity, we randomly sampled subnetworks of varying sizes from the observed GRNs and from one synthetic complete GRN, followed by a determination of their respective out-degree exponents (note here that sampled subnetworks do not have random degree connectivity, but are rather randomly sampled from GRNs). We discovered that for the observed GRNs, a majority of subnetworks exhibited exponents similar to the exponent of their respective GRNs (Fig 4). However, there exists a subnetwork size below which there is an increased uncertainty in the determination of the exponents. This is evident in the marked increase and overlap in variation of the subnetwork exponents across organisms at lower subnetwork sizes (Fig 4). To sample from synthetic networks, we first constructed *in silico* networks that capture the expected connectivity of the complete yeast GRN as prescribed by the predicted exponents and number of PDIs from the previous sections (see Methods for procedure on creating *in silico* GRNs). Expectedly, the out-degree distribution of the synthetic GRN was strikingly similar to the observed GRN (Fig 5A). Fitting power law functions on the out-degrees of synthetic GRN resulted in exponents ranging from 1.98 to 2.14, capturing the exponent (2.0) of the observed GRN (Fig 5B). Further, the power law fit to the out-degrees of subnetworks drawn from synthetic GRN was significant, as indicated by the large KS test *P* values (Fig 5C). In sharp contrast with previous studies that investigated properties of subnetworks by sampling nodes, rather than edges as done here, a majority of exponents of subnetworks were similar to the exponent of the complete GRN (Fig 5D), a further indication of organism-specific GRN connectivity. In addition, maintenance of the network connectivity in randomly sampled subnetworks demonstrated an important network property that distinguishes random network from scale-free networks: robustness. However, there is a subnetwork size threshold below which the organism-specific connectivity deviates from the expected. Our analysis revealed that whenever less than 10% of the complete *C*. *elegans* GRN; or 2% of the yeast and *Drosophila* GRNs are sampled, the expected scale-free property no longer holds (Fig 4).

Collectively, these observations have an important implication that is likely general to other scale-free GRNs: whenever subnetworks are randomly sampled from scale-free complete and sufficiently large incomplete GRNs that are either experimentally-determined or synthetic, the sampled subnetworks are scale-free, at least for a given subnetwork size threshold.

## Discussion

The ultimate goal in the characterization of TF-target gene interactions is to describe the complete genome-wide regulatory repertoire of an organism. However, current GRNs are incomplete. Two related challenges have impeded the understanding of an organism's complete regulatory repertoire: Establishing the full range of PDIs that characterize complete GRN, and anticipating the properties of complete GRNs given properties of experimentally-determined but incomplete GRNs, We have addressed these challenges by a topological analysis of current GRNs across a diversity of organisms, and discovered that observed GRNs, and their respective complete GRNs, have organism-specific topologies. This finding has profound biological implications: while GRNs are largely scale-free, there exists an organism-specific GRN architecture that drives organism-specific developmental trajectories and phenotypic uniqueness. Taking advantage of conservation of network topology between observed and complete GRNs, we predicted the possible range of PDI numbers for complete GRNs. Indeed, the forecasted complete GRN PDI numbers are just a fraction of the maximum number of PDIs that result when each TF binds all target genes. This observation deviates from the previously proposed continuous network model, whose fundamental property is that TFs have the potential to bind all genes in an organism [27]. For broader applications, our simulation method employed in estimation of the expected number of PDIs can be applied to different types of biological networks such as protein-protein interaction, protein phosphorylation, metabolic interactions, and genetic interaction.

In contrast with previous work by Stumpf and colleagues [25], we demonstrate here that subnetworks sampled from scale-free networks are scale-free. Several differences exist between our approach and the one previously published [25]. First, sampling nodes leads to a loss of out-degrees resulting in a deviation between out-degrees of the observed network and sampled subnetworks. In addition, sampling nodes leads to the generation of singletons (nodes without edges or targets) in the subnetworks. In contrast, we sampled edges (with their associated nodes) from observed GRNs, thereby capturing both a TF and its potential targets. Indeed, this approach mimics the expected experimental sampling. Second, stochasticity inherent to sampling procedures was not previously accounted for [25]. Here, we account for stochasticity in sampling procedure by repeated sampling of subnetwork and estimation of the variance of the sampling distribution of subnetwork exponents at each subnetwork size. Third, estimation of power law exponents using the graphical method of ordinary least squares (OLS, see Fig 2 of reference [25]) might not be a robust approach for parameter estimation. The OLS method is based on the following assumptions: (a) regression errors are identically and independently distributed (iid) random variables with mean zero, and (b) the standard deviation of the error is independent of the independent variable (out-degrees). The OLS method is expected to perform poorly in the estimation of the power law exponents because these assumptions are not met in empirical data of power law distributions [28, 29]. In our analysis, we fit the data (out-degrees of observed and sampled subnetworks) to the power law function using the maximum likelihood estimation (MLE) method, since MLE has been shown to be asymptotically efficient and can be applied to a wide range of data with skewed distributions [28].

Our study also provides an interpretation of the organism-specific power law exponent by use of Lorenz curves: GRNs with higher values of exponents are ‘egalitarian’ in their TF-target gene binding. Simply put, GRN architectures can either be ‘capitalistic’, exhibited by a highly skewed TF-target gene binding landscape described by low exponents; or ‘socialistic’, described by high exponents. Just like skewed distributions of incomes of individuals describe less egalitarian capitalist societies, we envisage a more skewed TF-target gene binding landscape in GRNs with comparatively low exponents, wherein the number of target genes bound by a TF is analogous to an individual’s income. An increase in the exponent value denotes a decrease in skewness of TF binding. Taken together, findings reported herein provide opportunity to understand complex regulatory mechanisms from a genome-wide perspective, while paving way for construction and analyses of GRNs in non-model organisms whose complete regulatory repertoire is yet to be deciphered.

## Methods

### Construction of observed GRNs

PDIs for *C*. *elegans*, *D*. *melanogaster*, *S*. *cerevisiae* and *A*. *thaliana* were extracted from regulatory databases and literature. In cases where regulatory interactions comprised of only DNA-binding sites (such as in ChIP-Seq, DAP-Seq, and ChIP-chip binding ‘peak’ location), the target genes associated with the binding sites were located within 2 kb of the ‘peak’ location. Transcriptional GRNs were subsequently modelled using directed graphs, *G*_*n*,*v*_, with *n* nodes and *v* vertices (edges, PDIs). Nodes in GRNs represent both target genes and their associated protein products in cases where a target gene is a TF. A PDI is represented by a directed edge emanating from the TF and ending in the target gene.

### Node degree and determination of scale-free property

Node degrees were determined by enumerating the number of TFs binding to each target gene (out-degree) and the number of target genes bound by each TF (in degree). A formal statistical framework that tests scale-free property in GRNs was developed involving the following steps:

(i) Fitting *n* node degrees to the power law function and estimating power law exponent using maximum likelihood approach. For a given minimum degree, *k*_*min*_, the scaling exponent is estimated by numerically optimizing the log-likelihood using the following maximum likelihood estimation function:
  $$\hat{\alpha }=1+n{\left[\sum_{i=1}^{n}log(\frac{k_{i}}{k_{min}-0.5})\right]}^{-1}$$ (8)
(ii) Determination of goodness-of-fit using the Kolmogorov-Smirnov (KS) distance metric, *D*; a measure of distance between the empirical degree distribution and the power law model fit defined by:
  $$D={max}_{k\geq k_{max}}|S(k)-P(k)|$$ (9)
  Here, *S(k)* denotes the empirical cumulative distribution function (CDF) and *P(k)* is the power law model. The *D* statistic is determined for values of *k* where *k≥*_*kmin*_. This implies that the estimate *k*_*min*_ is the value of *k* that minimizes the distance *D*. This is followed by the fit of each of the synthetic datasets on the power law model, and calculation of the KS *D* statistic between the empirical synthetic dataset and its corresponding power law model fit. The *p-*value is simply the fraction of times or datasets the KS statistic is larger than the KS statistic obtained from the observed data. Large *p-*values (typically greater than 0.1) suggest that the observed degrees are generated from a power law distribution.
(iii) Model selection using the non-nested likelihood ratio test. The Kullback-Leibler information criterion (Vuong’s closeness test) was employed in performing pairwise comparisons between significant power law model fits and other competing distribution models such as Poisson and Exponential distributions. The Vuong’s statistic tests the null hypothesis that models being tested are equally close to the true data generating process, with the alternative hypothesis that one model is closer. In the pair-wise comparison, a positive statistic and a *p-*value less than 0.05 denote that power law is closer to the data generating process.

Note that implementations of the methods presented above can be found in the R statistical packages ‘igraph’ and ‘poweRlaw’.

### Sampling subnetworks

Sampling subnetworks involved randomly selecting a number of PDIs from the observed GRNs, followed by construction of the subnetworks from the sampled PDIs. The sizes of the subnetworks correspond to the proportion of PDIs sampled. One thousand subnetworks were sampled for each proportion. A degree distribution was determined for each sampled subnetwork. Below is the pseudocode implemented for sampling and fitting power law function on the sampled subnetworks, from each GRN:

For *i* in {0,..,1} **//** where *i* is a proportion (size) of the GRN

    For *j* in {1,..,1000} **//** 1000 iterations

        Select edges uniformly at random from edge-list of GRN

        Construct subnet $G_{i,j}^{*}$

        Fit out-degree of subnet to Power law function

        Estimate exponent *α*_*i*,*j*_ of subnet out-degree

    END For

END For

To sample from synthetically-generated out-degrees, the following pseudocode was implemented:

Generate 10,000 degrees that follow a specified exponent (Note B in S1 Information)

For *i* in in {0,..,1} where *i* is a proportion of the number of TFs (degrees) in the GRN of interest

    For *j* in {1,…,1000} //1000 iterations

        Select j * i random TFs from the population

        Construct subnet $G_{i,j}^{*}$

        Fit $G_{i,j}^{*}$ out-degree to Power law function

        Estimate exponent *α*_*i*,*j*_

    END For

END For

### Simulating *in silico* GRNs

Estimation of the expected number of PDIs in complete GRNs, and the power law exponent alpha for observed GRNs, enabled creation of *in silico* GRNs that recapitulate the expected complete GRNs. Complete GRNs were built using the edited ‘igraph’ function ‘static_power_law_pl’ which takes exponent and number of PDIs as inputs. In order to generate a biologically comparative network, the number of TFs in the function ‘static_power_law_pl’ was edited so that only 5% of genes can have out-going edges. The scale-free property, sampling of subnetworks, and the determination of the clustering coefficients of the *in silico* GRNs were performed using methods outlined above.

### Evaluating the threshold/knee point of distribution parameter

The threshold where the exponents of samples start to deviate significantly is called the knee point of exponential function. A MATLAB code written by Dimitry Kaplan called Knee Point finds the knee point by fitting two lines (in each direction) at each bisection point and calculating the sum of errors of points along those lines. The knee is judged to be at the bisection point which minimizes the sum of errors of the two fits.

## Supporting information

S1 Information Contains Note A, Note B, and Supporting Tables.
(DOCX)

S1 Fig In-degree connectivity of gene regulatory networks. Histograms depicting in-degree distribution corresponding to GRNs for *D*. *melanogaster* (a), *C*. *elegans* (b), and *S*. *cerevisiae* (c). TG: target gene.
(EPS)

S2 Fig Sampling distribution of power law exponents. Distribution of exponents derived from large samples of synthetic degrees (n = 5000) is approximately normal (a), compared to smaller samples (b).
(EPS)

## References

[1] Hines P, Blumsack S, Sanchez CE, Barrows C, editors. The topological and electrical structure of power grids. The topological and electrical structure of power grids; 2010: IEEE.

[2] Barabási A-L, Oltvai ZN. Network biology: understanding the cell's functional organization. Nature reviews Genetics. 2004;5(2):101–13. doi: 10.1038/nrg1272 14735121 10.1038/nrg1272

[3] Barzel B, Barabási A-LL. Universality in network dynamics. Nature physics. 2013;9 doi: 10.1038/nphys2741 24319492 10.1038/nphys2741 PMC3852675

[4] del Sol A, O'Meara P. Small-world network approach to identify key residues in protein-protein interaction. Proteins. 2005;58(3):672–82. doi: 10.1002/prot.20348 15617065 10.1002/prot.20348

[5] Humphries MD, Gurney K, Prescott TJ. The brainstem reticular formation is a small-world, not scale-free, network. Proceedings Biological sciences / The Royal Society. 2006;273(1585):503–11. doi: 10.1098/rspb.2005.3354 16615219 10.1098/rspb.2005.3354 PMC1560205

[6] Lee TI, Rinaldi NJ, Robert F, Odom DT, Bar-Joseph Z, Gerber GK, et al Transcriptional regulatory networks in Saccharomyces cerevisiae. Science (New York, NY). 2002;298(5594):799–804. doi: 10.1126/science.1075090 12399584 10.1126/science.1075090

[7] Mejia-Guerra MK, Pomeranz M, Morohashi K, Grotewold E. From plant gene regulatory grids to network dynamics. Biochimica et biophysica acta. 2012;1819(5):454–65. doi: 10.1016/j.bbagrm.2012.02.016 22406342 10.1016/j.bbagrm.2012.02.016

[8] Pullen N, Jaeger KE, Wigge PA, Morris RJ. Simple network motifs can capture key characteristics of the floral transition in Arabidopsis. Plant signaling & behavior. 2013;8(11). doi: 10.4161/psb.26149 23989666 10.4161/psb.26149 PMC4106512

[9] Tuğrul M, Kabakçioğlu A. Anomalies in the transcriptional regulatory network of the yeast Saccharomyces cerevisiae. Journal of theoretical biology. 2010;263(3):328–36. doi: 10.1016/j.jtbi.2009.12.008 20004671 10.1016/j.jtbi.2009.12.008

[10] Yeger-Lotem E, Sattath S, Kashtan N, Itzkovitz S, Milo R, Pinter RY, et al Network motifs in integrated cellular networks of transcription-regulation and protein-protein interaction. Proceedings of the National Academy of Sciences of the United States of America. 2004;101(16):5934–9. doi: 10.1073/pnas.0306752101 15079056 10.1073/pnas.0306752101 PMC395901

[11] Albert R, Barabási AL. Statistical mechanics of complex networks. Reviews of modern physics. 2002.

[12] Dorogovtsev SN, Mendes JFF. Evolution of networks. Advances in Physics. 2002;51(4):1079–187. doi: 10.1080/00018730110112519

[13] Albert R. Scale-free networks in cell biology. Journal of cell science. 2005. 10.1242/jcs.02714 16254242

[14] Heyndrickx KS, de Velde VJ, Wang C. A functional and evolutionary perspective on transcription factor binding in Arabidopsis thaliana. The Plant …. 2014. 10.1105/tpc.114.130591 PMC4247581 25361952

[15] Shen-Orr SS, Milo R, Mangan S, Alon U. Network motifs in the transcriptional regulation network of Escherichia coli. Nature genetics. 2002 doi: 10.1038/ng881 11967538 10.1038/ng881

[16] Walhout AJ. Gene-centered regulatory network mapping. Methods Cell Biol. 2011;106:271–88. doi: 10.1016/B978-0-12-544172-8.00010-4 ; PubMed Central PMCID: PMCPMC3825173. 22118281 10.1016/B978-0-12-544172-8.00010-4 PMC3825173

[17] Mardis ER. ChIP-seq: welcome to the new frontier. Nat Methods. 2007;4(8):613–4. doi: 10.1038/nmeth0807-613 . 17664943 10.1038/nmeth0807-613

[18] O'Malley RC, Huang SC, Song L, Lewsey MG, Bartlett A, Nery JR, et al Cistrome and Epicistrome Features Shape the Regulatory DNA Landscape. Cell. 2016;166(6):1598 doi: 10.1016/j.cell.2016.08.063 . 27610578 10.1016/j.cell.2016.08.063

[19] Promislow D. A regulatory network analysis of phenotypic plasticity in yeast. Am Nat. 2005;165(5):515–23. doi: 10.1086/429161 . 15795849 10.1086/429161

[20] Schneider RF, Li Y, Meyer A, Gunter HM. Regulatory gene networks that shape the development of adaptive phenotypic plasticity in a cichlid fish. Mol Ecol. 2014;23(18):4511–26. doi: 10.1111/mec.12851 . 25041245 10.1111/mec.12851

[21] van Gestel J, Weissing FJ. Regulatory mechanisms link phenotypic plasticity to evolvability. Sci Rep. 2016;6:24524 doi: 10.1038/srep24524 ; PubMed Central PMCID: PMCPMC4834480. 27087393 10.1038/srep24524 PMC4834480

[22] Henshall JM, Tier B. An algorithm for sampling descent graphs in large complex pedigrees efficiently. Genet Res. 2003;81(3):205–12. . 12929911 10.1017/s0016672303006232

[23] Lee SH, Kim PJ, Jeong H. Statistical properties of sampled networks. Phys Rev E Stat Nonlin Soft Matter Phys. 2006;73(1 Pt 2):016102 doi: 10.1103/PhysRevE.73.016102 . 16486211 10.1103/PhysRevE.73.016102

[24] Leskovec J, Faloutsos C. Sampling from large graphs. Proceedings of the 12th ACM SIGKDD international conference on Knowledge discovery and data mining; Philadelphia, PA, USA. 1150479: ACM; 2006. p. 631–6.

[25] Stumpf MP, Wiuf C, May RM. Subnets of scale-free networks are not scale-free: sampling properties of networks. Proc Natl Acad Sci U S A. 2005;102(12):4221–4. doi: 10.1073/pnas.0501179102 ; PubMed Central PMCID: PMCPMC555505. 15767579 10.1073/pnas.0501179102 PMC555505

[26] Lorenz MO. Methods of measuring the concentration of wealth Publications of the American statistical association 1905 doi: 10.1080/152254371905.10503443.

[27] Biggin MD. Animal transcription networks as highly connected, quantitative continua. Dev Cell. 2011;21(4):611–26. doi: 10.1016/j.devcel.2011.09.008 . 22014521 10.1016/j.devcel.2011.09.008

[28] Bauke H. Parameter estimation for power-law distributions by maximum likelihood methods. The European Physical Journal B. 2007;58(2):167–73. doi: 10.1140/epjb/e2007-00219-y

[29] Ramsey F, Schafer D. The statistical sleuth: a course in methods of data analysis: Duxbury/Thomson Learning; 2002.

