# Supplementary material for: Topological and statistical analyses of gene regulatory networks reveal unifying yet quantitatively different emergent properties
Source: PLoS Comput Biol. 2018 Apr 30;14(4):e1006098. doi: 10.1371/journal.pcbi.1006098 (PMC5945062; doi:10.1371/journal.pcbi.1006098)
Supplement: S1 Information — Contains Note A, Note B, and Supporting Tables. (DOCX) [file pcbi.1006098.s001.docx]

**S1 INFORMATION**

**Note A: Sampling distribution of power law exponents and CLT**

The sampling distribution of power law exponents assumes a large population of out degrees (for instance *N ≥* 10,000 degrees) that are power law distributed with a population scaling exponent ***α_pop_***. Drawing ‘*r’* random and independent samples (with replacement) of size ‘*n’* results to variations of scaling exponents of each sample (*α_sample_*). Hence the sample scaling exponent becomes a random variable ***α_SAMPLE_*** with:

$\alpha_{SAMPLE}=\{\alpha_{1}, \alpha_{2}, \alpha_{3},..,\alpha_{r} \}$ (1)

where *α_1_...α_r_* are value of *α_sample_* associated with *r* samples. The expected value of the random variable ***α_SAMPLE_*** can be obtained by averaging over sample exponents:

$E\left[ \alpha_{SAMPLE} \right]=\alpha_{SAMPLE,avg}=\frac{1}{r}\sum_{i-1}^{r} \alpha_{i}$. (2)

It follows that:

$\alpha_{SAMPLE,avg}=\alpha_{pop}$; for large *n*. (3)

Since on average we expect ***α_sample_*** to equal ***α_pop_***, and since the distribution of the random variable $\alpha_{SAMPLE}$is approximately normal with mean $\alpha_{SAMPLE,avg}$ and standard deviation ***σ*** (Fig S2a), then according to the central limit theorem (CLT), $\alpha_{SAMPLE}$ is an unbiased estimate of $\alpha_{pop}$. The CLT therefore enables derivation of 95% prediction intervals (PIs) of the observed exponent $\alpha_{obs}$ by:

$PI=\alpha_{obs}\pm t_{n-1, 0.975}^{*}\times s\times\sqrt{1+1/r}$ (4)

where *t**  is the Student’s t critical value at a specified two-sided confidence level, and *s* is the standard deviation of exponents derived from a population of synthetic degrees generated from a Monte-Carlo simulation (see S1 Note 2).

**Note B: Estimation of the expected number of PDIs for complete GRNs**

Given that the observed exponents are estimates of the complete GRN out-degree distribution, we posit that the number of PDIs for a complete GRN can be calculated by:

$I_{comp}=\sum_{i=1}^{N} k_{comp,i}$ (5)

where *N* is the total number of TFs in the genome and *k_comp,i_* is the *i^th^* simulated out degree of the theoretically complete GRN. We generate degree *k_comp,i_* from a power law probability mass function (pmf) with an exponent corresponding to the estimated exponent ($\alpha_{obs}$), with:

$k_{comp,i}=k_{u}^{-1/(\alpha_{obs}-1)}$ (6)

where *k_u_* is a degree sampled from a uniform distribution with the density:

$f\left( k_{u} \right)=1/{(max-min)}$; for *min*$\leq$*k_u_* $\leq$ *max*  (7)

Thus, obtaining a summation of all values of *k_comp,i_* yields the number of interactions for complete GRNs, as depicted in equation 5. Two constraints are applied to this simulation: (i) the lower and upper bounds of the simulated degrees are defined by the 95% PIs of the exponent (equation 4); and (ii) the maximum value for out-degree approaches but is less than the number of target genes in the genome.

To determine the accuracy of the simulation approach, we investigated how well the proposed method estimates the number of interactions for the observed GRNs (*I_obs_*). When *n =* 10,000 iterations of simulations — comprising derivation of degrees from a power law pmf with specified exponent, and obtaining their associated number of interactions by degree summation — are performed for a given observed GRN, the result is a vector *I_EST_* of length 10,000 whose elements are the number of estimated interactions for each iteration, that is:

$I_{EST}=\{I_{est,1}, I_{est,2}, \ldots, I_{est,n}\}$ (8)

and

$I_{EST,avg}=E\left( I_{EST} \right)=\sum_{i=1}^{n} I_{est,i}$ (9)

where *I_EST,avg_* is the average number of PDIs for *n* iterations of simulation.

We employ a classical hypothesis testing procedure and test the difference between the observed number of interactions (*I_obs_*) and the predicted number of interactions (*I_EST,avg_*) for the observed GRNs (note that *I_EST,avg_* is not the predicted number PDIs for the complete GRN), with:

$H_{0}:I_{obs}$ *=*$I_{EST,avg}$

$H_{1}:I_{obs}$ *≠* $I_{EST,avg}$

We test the difference between *I_obs_* and *I_EST,avg_* using a *Z-*score:

$Z=(I_{EST,avg}-I_{obs}/s)$ (10)

**Supporting Tables**

**Table A: *D. melanogaster* connectivity metrics for data source-specific subnetworks.** Note that a summation of number of interactions across different data source types does not equal to the number of interactions for the network with all data because of redundancy. That is, an interaction identified in two techniques is represented once in the network incorporating data from all techniques. NA: not available value because of non-power law degrees.

| **Data source** | **# of interactions** | **Exponent**  **(KS *P-*value)** | **# of nodes**  **(# of nodes that are TFs)** |
| --- | --- | --- | --- |
| ChIP-Seq; ChIP-chip; Y1H | 229,615 | 3.04  (0.82) | 15,302  (166) |
| ChIP (*i.e.* ChIP-Seq; ChIP-chip) | 229,264 | 3.04  (0.82) | 15,286  (59) |
| ChIP-chip | 65,943 | 3.08  (0.97) | 13,888  (22) |
| ChIP-Seq | 166,117 | 3.36  (0.57) | 14,751  (38) |
| Y1H | 406 | NA | 208  (136) |
| Embryo-derived interactions | 178,224 | 3.10  (0.86) | 15,016  (47) |

**Table B*: C. elegans* connectivity metrics for data source-specific subnetworks.** Available data for *C. elegans* was ChIP-Seq and Y1H. The exponent obtained from including only ChIP-Seq experiments matches the expected exponent of the observed network. In the Y1H subnetwork, one TF binds on average one target gene (225/169), hence the lack of scale-free connectivity (row number 4). NA: not available value because of non-power law degrees.

| **Data source** | **# of interactions** | **Exponent**  **(KS *P-*value)** | **# of nodes**  **(# of nodes that are TFs)** |
| --- | --- | --- | --- |
| ChIP-Seq; Y1H | 464,258 | 4.12  (0.700) | 20,220  (219) |
| ChIP-Seq | 463,618 | 4.25  (0.80) | 20,213  (67) |
| Y1H | 667 | NA | 225  (169) |

**Table C: *S. cerevisiae* connectivity metrics for data source-specific subnetworks.**

| **Data source** | **# of interactions** | **Exponent**  **(KS *P-*value)** | **# of nodes**  **(# of nodes that are TFs)** |
| --- | --- | --- | --- |
| ChIP; Y1H | 26,091 | 2.00  (0.50) | 6,072  (151) |
| Y1H | 1079 | 2.4  (0.99) | 688  (131) |

**Table D: Pairwise comparisons of empirical out degrees of observed GRNs using the Kolmogorov-Smirnov (KS) test.** D is the KS statistic distance measure between two empirical distributions of data, and alpha threshold is 0.1

|  | ***A. thaliana*** | ***D. melanogaster*** | ***C. elegans*** | ***S. cerevisiae*** |
| --- | --- | --- | --- | --- |
| ***A. thaliana*** | X | D: 0.02  *P*-value: 0.007 | D: 0.03  *P*-value: 0.000 | D: 0.02  *P*-value: 0.318 |
| ***D. melanogaster*** | X | X | D: 0.01  *P*-value: 0.043 | D: 0.02  *P*-value: 0.137 |
| ***C. elegans*** | X | X | X | D: 0.03,  *P*-value: 0.001 |
